# Retraction: Biogenic synthesis of ZnO and Al_2_O_3_ nanoparticles using *Camellia sinensis* and *Origanum vulgare* L. leaves extract for spectroscopic estimation of ofloxacin and ciprofloxacin in commercial formulations

**DOI:** 10.1371/journal.pone.0360347

**Published:** 2026-10-12

**Authors:** The PLOS One Editors

After this article [1] was published, concerns were raised regarding results presented in Figs 1 and 5.

Specifically:

- The following panels appear to overlap despite representing different experimental results:
  - Fig 1c in [1] and Fig 2b in [2].
  - Fig 5a in [1], Fig 3a in [2], and Fig 2a in [3, retracted in 4].
  - Fig 5c in [1] and Fig 3a in [5].
- There appear to be repetitive elements in the background within the following panels:
  - Fig 5b.
  - Fig 5c.
  - Fig 5d.
- In Figs 1c and 1d, some of the wavenumber markers do not match the corresponding positions on the x-axis.
- In Fig 1d, there appear to be areas of discontinuity in the spectral trace.

The corresponding author provided data underlying Figs 1 and 5. Upon editorial assessment, these data did not resolve the above concerns.

In light of the unresolved concerns that question the validity and integrity of the reported results, the *PLOS One* Editors retract this article.

MFET did not respond to the final editorial decision. NAA, HAA, WAAO, and AMAM either did not respond directly or could not be reached.
